# The cellular 3D printer of a marine bristle worm—chaetogenesis in *Platynereis dumerilii* (Audouin & Milne Edwards, 1834) (Annelida)

**DOI:** 10.1007/s00441-022-03731-9

**Published:** 2022-12-23

**Authors:** Ekin Tilic, Tim Herkenrath, Gregor Kirfel, Thomas Bartolomaeus

**Affiliations:** 1 Institute of Evolutionary Biology and Animal Ecology, Rheinische Friedrich-Wilhelms-Universität Bonn, An der Immenburg 1, Bonn, 53121 Germany; 2 Marine Biological Section, Department of Biology, University of Copenhagen, Copenhagen, Denmark; 3 Institute of Cell Biology, Rheinische Friedrich-Wilhelms-Universität Bonn, Ulrich-Haberland-Str. 61a, Bonn, 53121 Germany

**Keywords:** Polychaeta, Chitin, Ultrastructure, Morphogenesis, Microvilli

## Abstract

Annelid chaetae are extracellular chitinous structures that are formed in an extracellular epidermal invagination, the chaetal follicle. The basalmost cell of this follicle, the chaetoblast, serves like a 3D-printer as it dynamically shapes the chaeta. During chaetogenesis apical microvilli of the chaetoblast form the template for the chaeta, any structural details result from modulating the microvilli pattern. This study describes this process in detail in the model organism *Platynereis dumerilii* and clarifies some aspects of chaetogenesis in its close relative *Nereis vexillosa*, the first annelid in which the ultrastructure of chaetogenesis had been described. Nereid species possess compound chaetae characteristic for numerous subgroups of errant annelids. The distal most section of these chaetae is movable; a hinge connects this part of the chaeta to the shaft. Modulation of the microvilli and differences in their structure, diameter and number of microvilli, and their withdrawal and reappearance determine the shape of these compound chaetae. Chaetal structure and pattern also change during life history. While larvae possess a single type of chaeta (in addition to internal aciculae), juveniles and adults possess two types of chaetae that are replaced by large paddle-shaped chaetae in swimming epitokous stages. Chaetogenesis is a continuous process that lasts during the entire lifespan. The detailed developmental sequence of chaetae and their site of formation are very similar within species and species groups. We expect that similarity results from a conserved gene regulatory network making this an optimal system to test the phylogenetic affinity of taxa and the homology of their chaetae.

## Introduction

Chaetae, the bristles of bristle worms, are one of the most characteristic features of Annelida, a metazoan taxon consisting of roughly 22.000 species (Rouse et al. 2022). Chaetae aid the animals in locomotion, moving substrate, nutrition, defense, and relocating within and holding on to tubes or burrows they live in (Merz and Edwards 1998; Merz and Woodin 2000; Woodin et al. 2003; Merz 2015). The importance of chaetae strongly influences the traditional but now outdated classification of annelids according to the number of their eponymous bristles, namely as polychaetes or as oligochaetes (Fauchald and Rouse 1997; Bartolomaeus et al. 2005; Weigert and Bleidorn 2016; Capa and Hutchings 2021; Rouse et al. 2022). In 1973, Gustus and Cloney published a detailed description of the compound (jointed) chaetae of *Nereis vexillosa* Grube, 1851 and a year later, they published their paper on chaetogenesis in *Nereis vexillosa* (Gustus and Cloney 1973; O’Clair and Cloney 1974). These papers demonstrated the complexity and intricacy of chaetal formation and the involvement of multiform and dynamic microvilli. Even today, their studies strongly influence our current knowledge on the process of chaetal formation (Warren 2015).

Annelid chaetae are extracellular chitinous structures that are formed in an epidermal invagination, the chaetal follicle. Microvilli emanate from the basalmost cell into the lumen of the follicle, visible at high magnification in histological sections (Bobin 1944; Bouligand 1966, 1967). This basalmost cell, the chaetoblast, functions almost like a 3D-printer as it shapes the structure of the chaeta by modulating the pattern of its apical microvilli while continuously releasing N-acetylglucosamine (Gustus and Cloney 1973; O’Clair and Cloney 1974). Since these molecules continuously polymerize to chitin between the microvilli, the fully differentiated chaetae are nothing but these modulations of the apical microvilli pattern frozen in time. During this process, as a chaeta elongates, numerous empty compartments mark the former position of the microvilli. These compartments cause a characteristic hexagonal pattern of chitin deposition in transversely sectioned chaetae (Hausen 2005).

Chaetae are without doubt one of the most studied morphological structures of annelids. Within Annelida, chaetae have a remarkable morphological diversity and are often species- and/or taxon specific (Tilic et al. 2016). Their enormous diversity primarily depends on the dynamics of the apical microvilli of the chaetoblast. Recent studies, however, showed that cellular interaction within and among chaetal follicles strongly influence the shape of the chaetae (Bartolomaeus 1998; Hausen 2005; Tilic et al. 2014, 2015; Tilic and Bartolomaeus 2016). These follicular interactions correlate with chaetal follicles that are arranged in a row running perpendicular or oblique to the body axis. Within each row, chaetogenesis happens on the one end, whereas chaetal degeneration occurs on the opposite side (Bartolomaeus 1998). Certain annelid species, like *Nereis vexillosa*, possess complex compound chaetae consisting of moveable apical sections. Since their chaetae are not arranged in a row, but in a bundle, it remains to be tested whether interfollicular interactions within a bundle also influence the shape of these chaetae.

According to our present knowledge on chaetogenesis, however, details of chaetogenesis as provided by Gustus and Cloney (1973) and O’Clair and Cloney (1974) must result from erroneous interpretation. Especially the formation of a comb-row of teeth characteristic for the movable blade and also the ambiguities in hinge development call for revisiting chaetogenesis of compound chaetae. In this paper we provide a detailed description of chaetogenesis in *Platynereis dumerilii* and revisit O’Clair and Cloney’s (1974) reconstruction of chaetogenesis of nereidid compound chaetae. *Platynereis dumerilii* is one of the most prominent model annelids with a well-studied development and reproduction (Özpolat et al. 2021). It can be bred and cultured relatively easy in aquaria and has a lifelong proliferation of homonomous segments, rendering it an ideal model to study annelid development, and the molecular cascades involved in segmentation and chaetal formation (Fischer and Dorresteijn 2004; Zakrzewski 2011; Gazave et al. 2017; Kuehn et al. 2019). Using serial transmission electron microscopy, confocal imaging, histology, and 3D reconstruction, we investigated the chaetal topology and arrangement of formative sites in addition to chaetal ultrastructure and chaetogenesis in different life history stages of this model organism.

## Materials and methods

### Animals

**Fig. 1:**
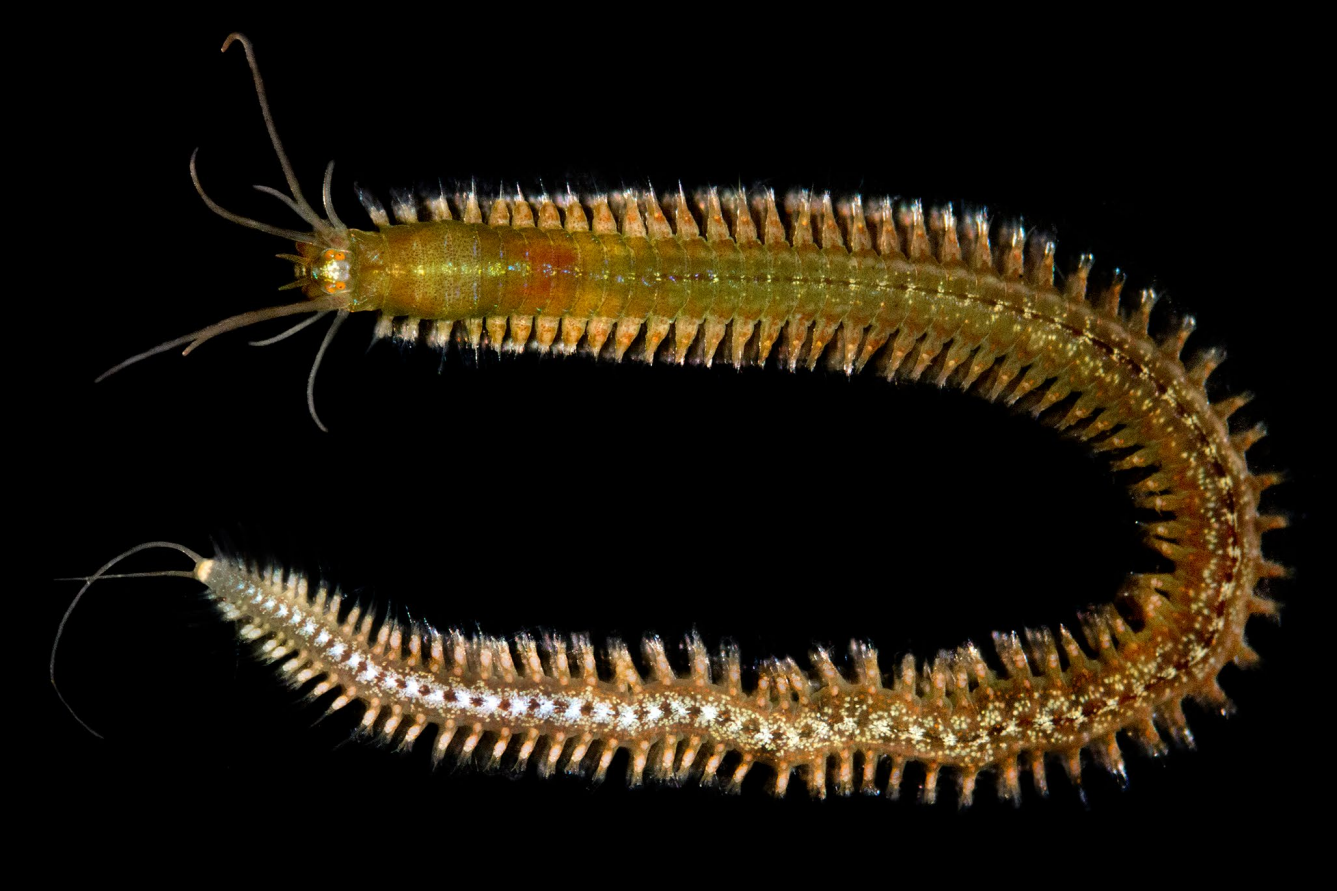
*Platynereis dumerilli* (Audouin & Milne Edwards, 1833) live habitus of an atokous individual

All specimens of *Platynereis dumerilii* (Audouin & Milne Edwards, 1833) (Fig. 1) investigated in this study were obtained from an 18 °C breeding culture established in the Institute of Evolutionary Biology, Bonn (COI sequence available at NCBI GenBank Accession number: MH114981). The animals studied were 48-h-old metatrochophora stages, 72-h and 96-h-old nectochaeta stages, 30-day-old juveniles, and epitokous stages.

### Histology and transmission electron microscopy (TEM)

The specimens used for semi-thin histology and for TEM (transmission electron microscopy) were fixed with 2.5% glutaraldehyde buffered in 0.05 M phosphate buffer. Ruthenium red was added to the fixative. The animals were directly dissected into the fixative and fixed for 1 h at 4 °C. The samples were then rinsed in the same buffer and stored in a buffer containing NaN_3_ until embedding. Prior to dehydration in an ascending acetone series and embedding in araldite, the specimens were postfixed in 1% OsO_4_ buffered in PBS for 30 min (at 4 °C). The posterior end of the juvenile animal (7 praepygidial segments), a fully differentiated parapodium of an anterior segment, and an epitokous segment were sectioned into a complete series of semi-thin sections (1 µm) using a Diatome Jumbo diamond knife in a Leica Ultracut S microtome, following the method described by Blumer et al. (2002). The semi-thin sections were stained with toluidine blue, covered with a coverslip mounted with araldite, analyzed with an Olympus microscope (BX-51), and photographed with an Olympus camera equipped with the dot.slide sytem (2.2 Olympus, Hamburg). Images were aligned using IMOD (Boulder Laboratories, Kremer et al. 1996) and IMOD-align (http://www.evolution.uni-bonn.de/mitarbeiter/bquast/software). Aligned histological sections were used to reconstruct the 3D model of chaetal topology and the chaetal formation in the posterior-most segments.

A complete series of silver-interference colored (70–75 nm) ultra-thin sections were prepared of both metatrochophora stages, the nectochaeta and of the chaetal formative sites in the juvenile and in an epitokous segment. The ultra-thin sections were prepared with a diamond knife (Diatome) on a LEICA U6 ultramicrotome and placed on Formvar-covered, single-slot copper grids. They were stained with uranyl acetate and lead citrate in an automated TEM stainer (QG-3100, Boeckeler Instruments). Sections were examined using a ZEISS Libra 120 electron microscope equipped with the TRS camera system and a ZEISS EM 10 with phosphor imaging plates (Ditabis).

### 3D modeling

3D models of chaetal topology were generated using the software 3DsMax 13. Aligned histological images were imported as surface materials and the chaetae were modeled using standard cylindrical objects. When necessary, these were modified as NURBS (Nonuniform rational B-Splines)-surfaces.

### Confocal laser scanning microscopy (CLSM)

Animals used for CLSM (confocal laser scanning microscopy) were relaxed using MgCl_2_ and fixed in 4% paraformaldehyde (1 h, 4 °C). Single segments were later dissected to isolate parapodia. Isolated parapodia and segments were permeabilized with 0.1% Triton X-100 (Fisher Scientific). The parapodia were stained overnight at 4 °C with TRITC–phalloidin at a dilution of 1:100. After staining, parapodia were rinsed in PBS buffer. Samples were directly placed in hollow-ground slides and quickly dehydrated in an isopropanol series, cleared and mounted with Murray Clear (BABB).

### SEM

Animals used for SEM (scanning electron microscopy) were relaxed using MgCl_2_ and fixed using Bouin’s fluid for 24 h, transferred into 70% ethanol and washed therein twice. The specimens were then kept in a 0.5% phosphotungstic acid solution in 70% ethanol for 2 h. All samples were dehydrated in an ascending alcohol series and were dried in a critical point dryer (BALZERS) with CO_2_. After dehydration, the samples were sputtered with gold and platin (BALZERS sputter coater) and examined using an XL30 SFEG (Philips Electron Optics) and an FEI Verios 460L scanning electron microscope.

## Results

**Fig. 2:**
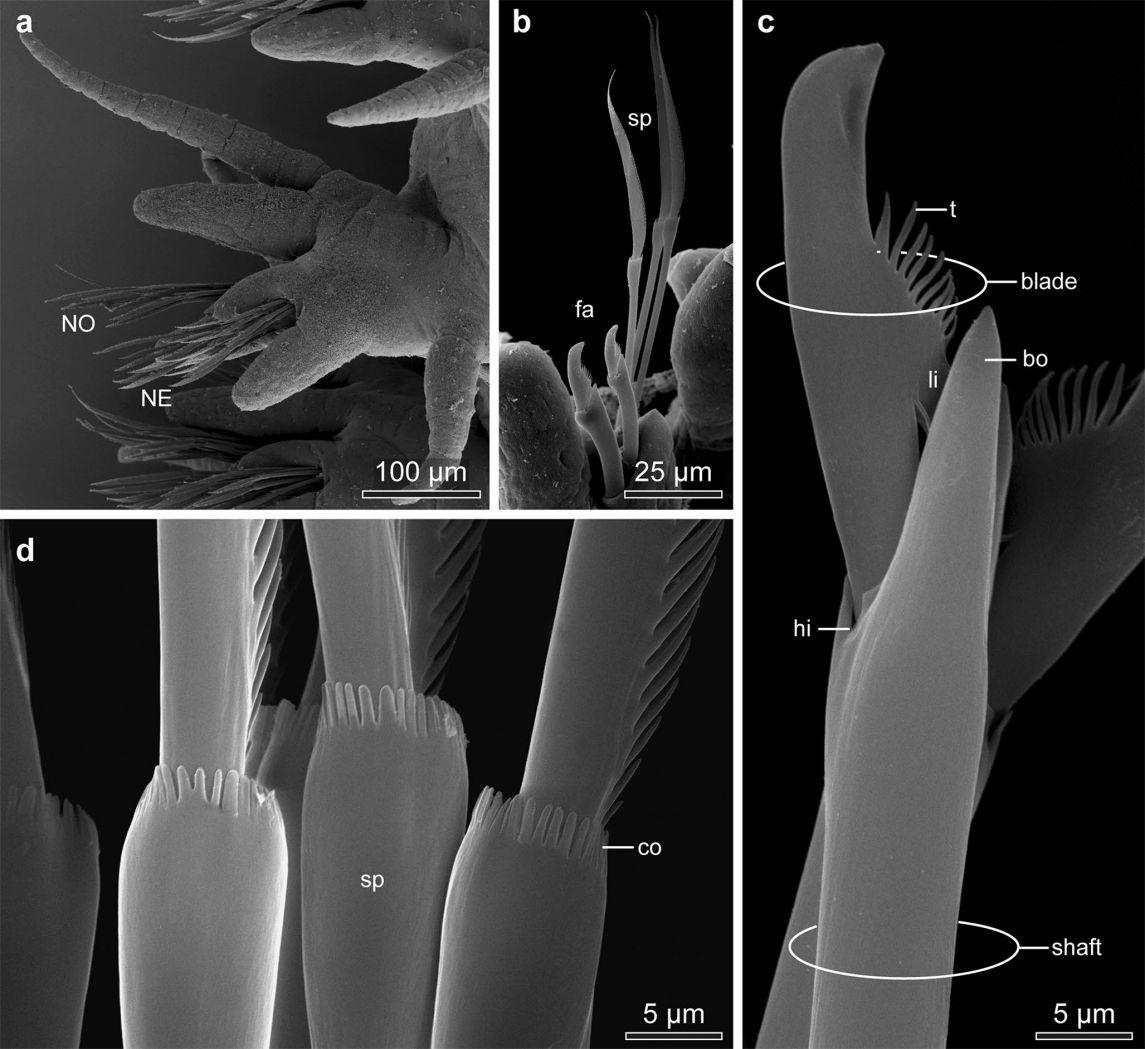
SEM micrographs of *Platynereis dumerilii* chaetae. **a** Overview of a parapodium, with the ventral neuropodium (*NE*) and dorsal notopodium (*NO*). **b** Chaetae from a posterior (younger) segment with only two notopodial falcigers (*fa*) and three neuropodial spinigers (*sp*). **c** Detail of a falciger, the movable portion of the chaeta (*blade*) has an adrostral rim armored with teeth (*t*) and is joined to the *shaft* with the single flexible ligament (*li*). The adrostral edge of the hinge (*hi*) is called boss (*bo*). **d** Detail of the collar region (*co*) of a spiniger (*sp*)

**Fig. 3:**
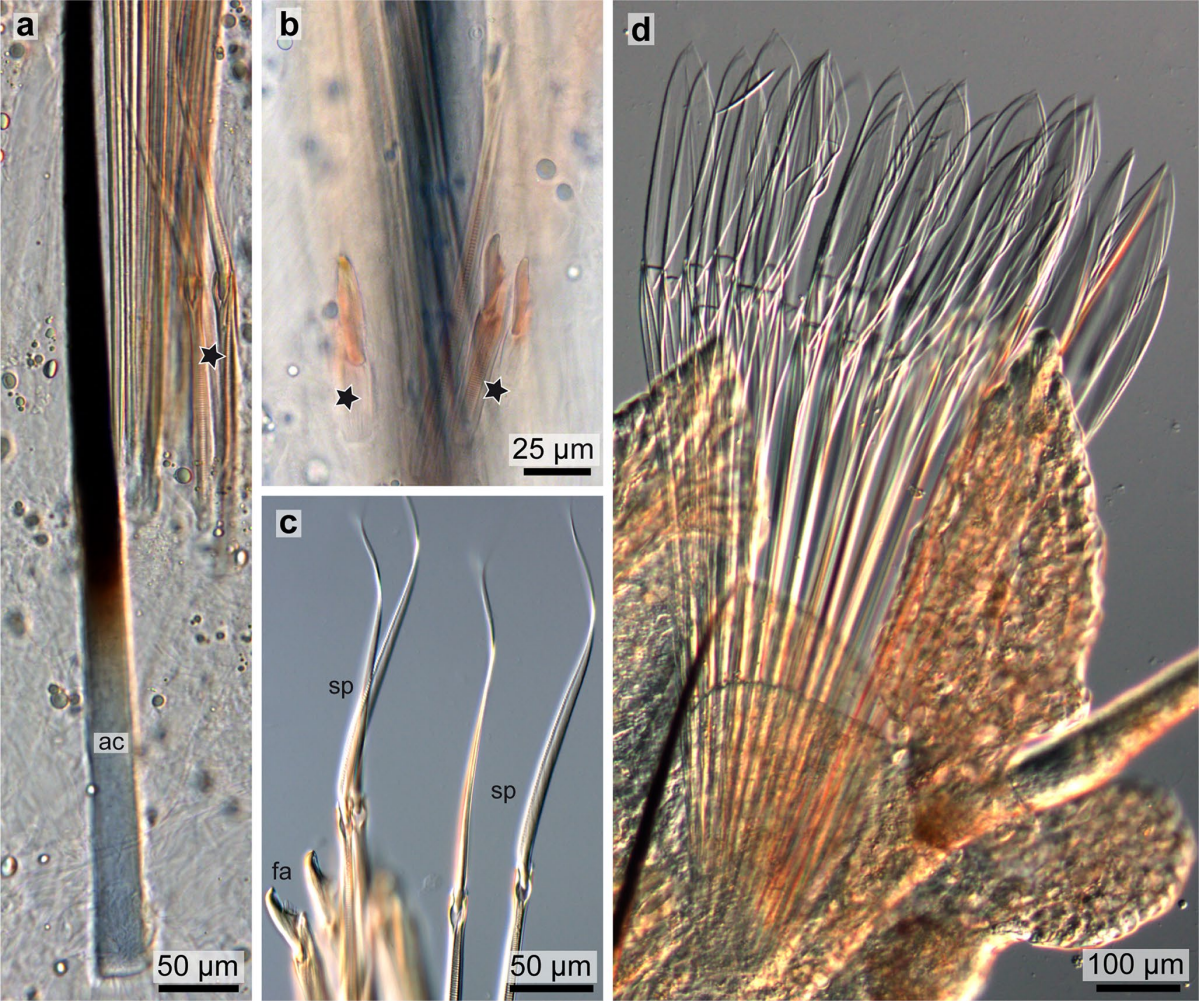
Light micrographs of *Platynereis dumerillii* chaetae. **a** A notopodial acicula (*ac*) and dorsally located developing spinigers (*star*), note the changing coloration of the acicula towards the base, and the lack of a central septated canal. **b** Developing falcigers marked with *stars*. **c** Blades of falcigers (*fa*) and spinigers (*sp*). **d** Paddle-like chaetae of an epitokous stage

In *Platynereis dumerilii* body segments are largely homonomous (Fig. 1). Each segment has biramous parapodia with prominent parapodial lobes and cirri (Fig. 2a). Notopodia and neuropodia are digitiform (Fig. 2), and both are reinforced by a single acicula (Fig. 3a, d). In addition to the aciculae, two different types of chaetae are present, falciger and spiniger chaetae (Figs. 2b and 3c). Both chaetae can be classified as compound chaetae, as a joint connects their blade-like distal section, simply called “blade” by O’Clair and Cloney (1974), to the shaft (Fig. 2c).

Notochaetae were almost exclusively spinigers (Figs. 2b and 3c); falcigerous chaetae only occured in the notopodia of the posterior segments. Neuropodia bore spinigers as well as falcigers (Fig. 3c).

During maturation both sexes of *Platynereis dumerillii* go through a sexual metamorphosis. In the epitokous, sexually mature stage, also called heteronereis, regular chaetae (falcigers and spinigers) get replaced by chaetae with paddle-like blades (Fig. 3d).

### Arrangement of the chaetae

**Fig. 4:**
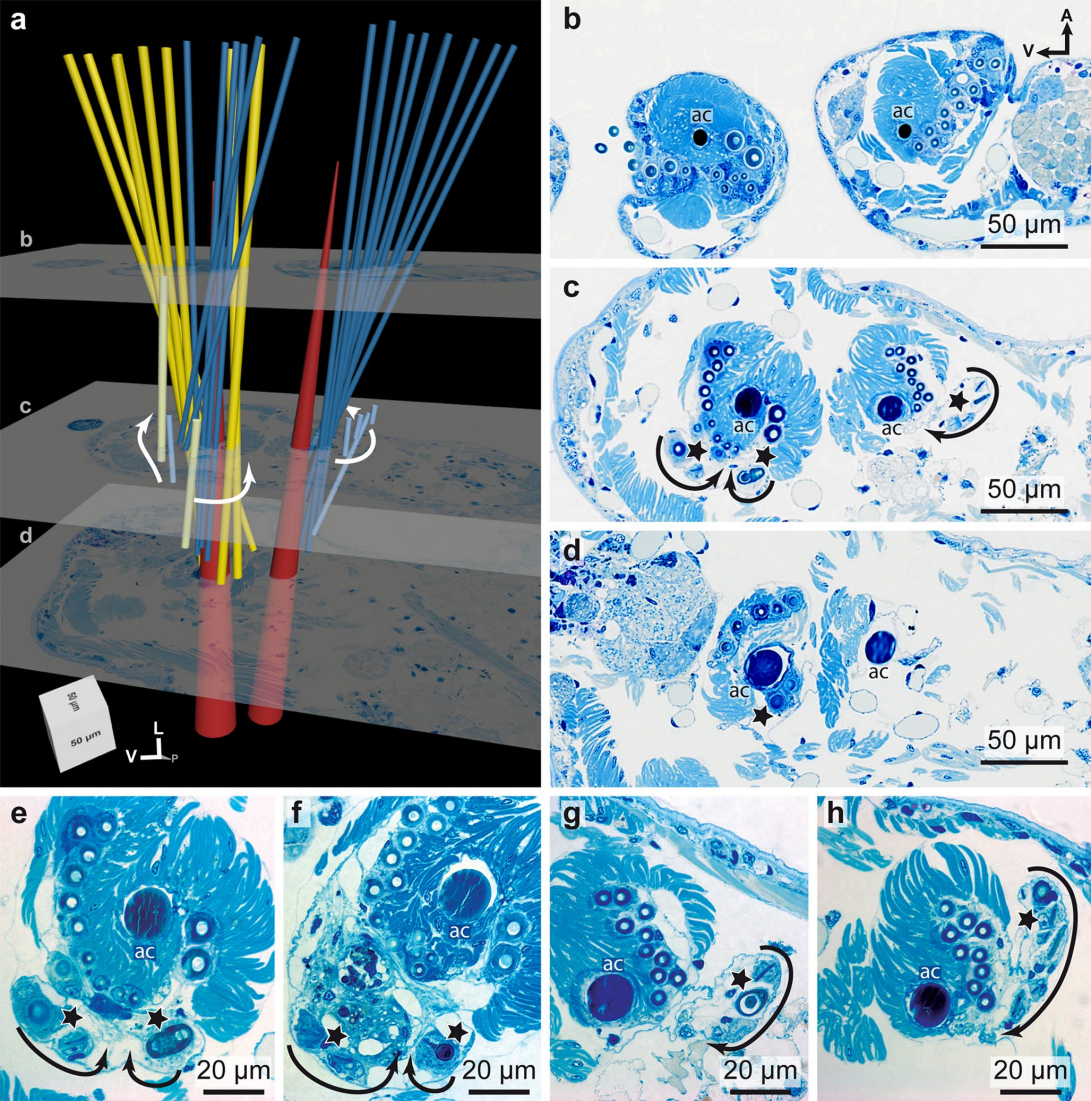
3D model and serial histological sections of a parapodium. **a** Spinigers are shown in blue, falcigers in yellow, aciculae (*ac*) in red. Histological sections are shown in **b**–**d** and their corresponding positions are marked in (**a**). **e**, **f** Neuropodial formative sites (marked with *stars*). **g**, **h** Notopodial formative site. *Arrows* indicate the direction of chaetae formation. The body axis is marked in a and b for orientation. All histological sections have the same orientation. *L* lateral, *V* ventral, *P* posterior, *A* anterior. The aligned serial sections of the parapodium can be downloaded at: https://doi.org/10.5281/zenodo.6957608

The notochaetae in *Platynereis dumerilii* were arranged in a single supra-acicular bundle, whereas the neurochaetae appeared to be separated in two groups, a supra- and a sub-acicular bundle (Fig. 4a). The ventral, sub-acicular group of neurochaetae only consisted of falcigers whereas the supra-acicular bundle mainly consisted of spinigers with the exception of a few (mostly 2) falcigers. Notochaetae and neurochaetae arose from a single chaetal sac respectively. Musculature attached to the chaetal sac deeper inside the tissue and since during fixation the muscles become fixed at a certain stage of contraction, chaetae twist and appear in a coiled arrangement that spirals around the acicula (Fig. 4).

Chaetogenesis occurs continuously in each chaetiger of *Platynereis dumerilii* (Fig. 3a, b). The site of chaetal formation was isolated in noto- and neuropodia (Fig. 4c). Within the notopodial chaetal sac there was only one site of chaetal formation, located at the dorsal edge and resembling a pouch that bulges out of the chaetal sac (Fig. 4g, h).

Neurochaetae however, arose from two separate formative sites; one giving rise to the sub-acicular bundle, the other to the supra-acicular one (Fig. 4a). The developmental sites of the neuropodium were located on the frontal edge of the chaetal sac (Fig. 4e, f).

All developmental sites were located at the same height along the distoproximal axis within the parapodium (Fig. 4a, c). The formation of new bristles appears to occur within the formative zone in a mirrored S-curve. It begins at the dorsal border of the formative site, approaches the acicula at first, but again moves away until it is positioned above the acicula above the remaining fully differentiated chaetae.

### Chaetal musculature

**Fig. 5:**
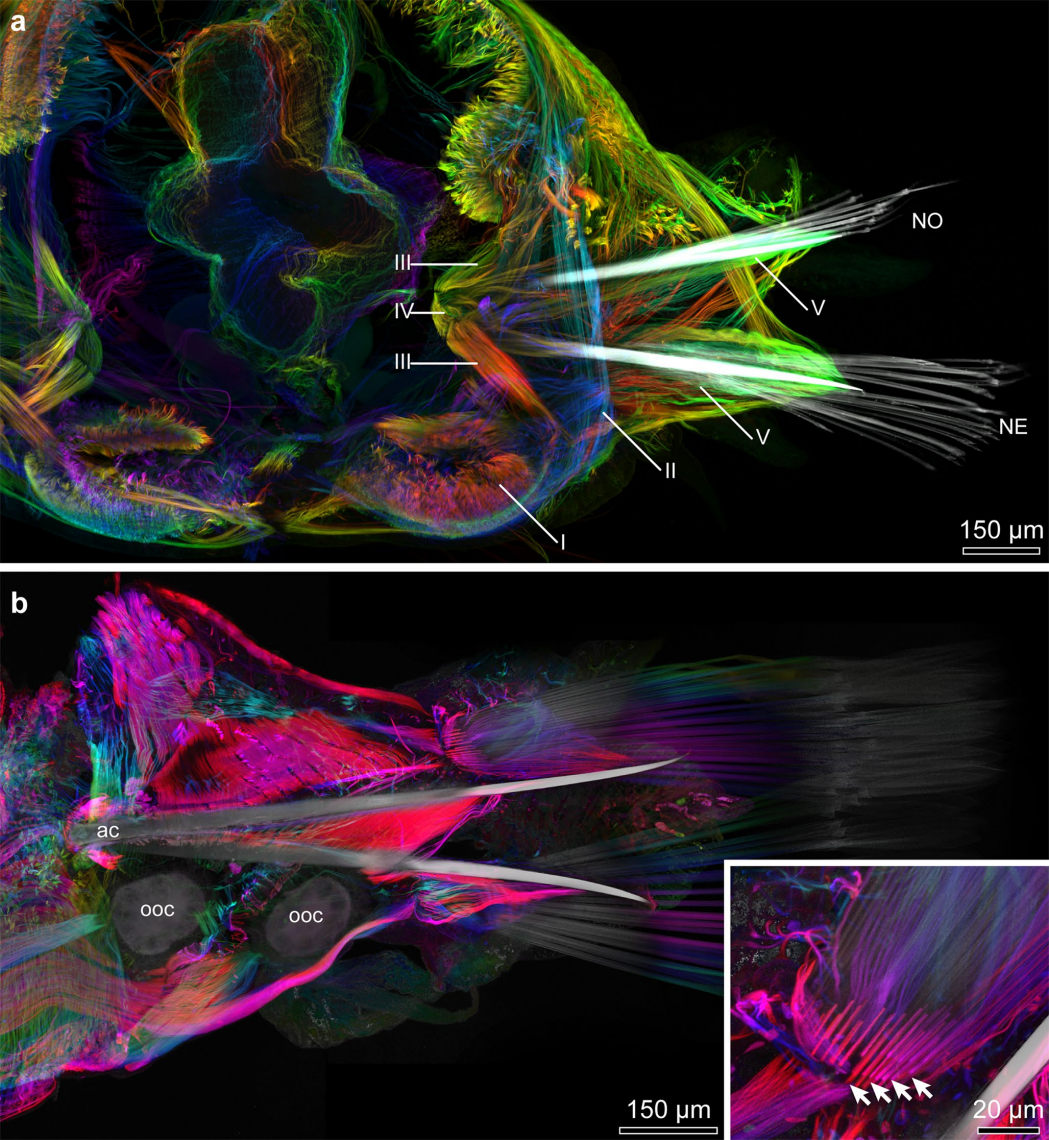
Depth coded confocal z-stack projections of disseted and phalloidin stained parapodia. **a** Parapodium of an atokous individual, with the dorsal notopodium (*NO*) and ventral neuropodium (*NE*). Roman numerals mark the major muscle groups. *I* thick bands of longitudinal body-wall musculature, *II* thin layer of ring musculature of the body-wall, *III* acicular muscles, *IV* short muscle band connecting the two aciculae, *V* muscles attaching to the neuropodial and notopodial chaetal sacs. **b** Parapodium of an epitokous female, with large oocytes (*ooc*) in the coelomic cavity. Note the stronger acicular musculature, attaching to a much larger portion of the acicula and not just its base. The inlet shows detail of the bases of paddle-like chaetae with > 20 µm long microvilli of the chaetoblast still visible within the chaetal shaft

An intricate network of chaetal and parapodial musculature control the movement of aciculae and groups of chaetae (Fig. 5). Large and robust aciculae that insert deep inside the animal’s body serve as an attachment site for prominent acicular musculature. These acicular muscles originated laterally from the body wall and inserted at the basis of the aciculae (Fig. 5a, III). Another short bundle of musculature connected both aciculae with one another (Fig. 5a, IV). The remaining chaetal musculature originated from the aciculae and attached to the extracellular matrix that surrounds the chaetal sac. Hence, functioning as protractors/retractors, and pushing the chaetal bundles outwards and inwards. The controlled movement of single chaeta is not possible.

During metamorphosis into the epitokous stage, the parapodial and body wall musculature underwent dramatic changes and the muscle bundles that connected to aciculae drastically increased in size (Fig. 5b). Staining of actin filaments with Phalloidin also allowed visualizing the dynamic microvilli that insert into a chaeta at its base (Fig. 5b). In the paddle-shaped chaetae of epitokous stages, these microvilli were extremely long reaching more than 20 µm into a chaeta.

### Formation of noto- and neuropodia

**Fig. 6:**
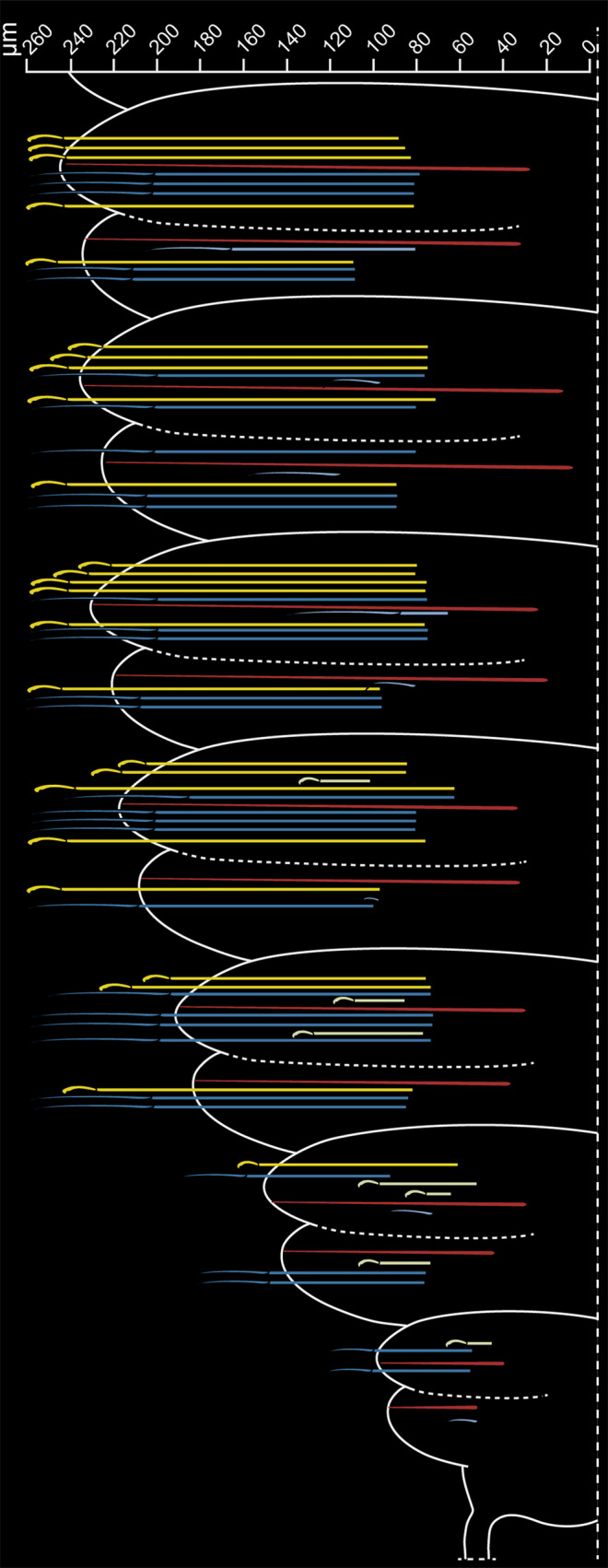
Schematic illustration of chaetal development in the posteriormost chaetigers. Aciculae are shown in *red*, spinigers in *blue*, and falcigers in *yellow*. The aligned serial sections of the posterior segments can be downloaded at: https://doi.org/10.5281/zenodo.6957628

Chaetal formation was inferred from several developmental stages in the posterior-most segments. The parapodia of the praepygidial segments were not fully differentiated, and the analysis of several chaetigers in a posterio-anterior axis revealed many details on the order in which certain chaetal types are formed. In order to illustrate chaetal formation in the posterior segments of *Platynereis dumerilii*, we investigated an aligned series of sagittal semi-thin sections through seven segments anterior to the growth zone (Fig. 6).

All segments, even the posteriormost (youngest) segment, bore internal aciculae that inserted deep inside the worm’s body. This segment bore only a few other chaetae in addition to aciculae; one developing spiniger in the notopodium, two spinigers, and one developing falciger in the neuropodium. In contrast to older, fully differentiated anterior segments, both chaetal types were present in the notopodium of the posterior segments. The formative sites in the posterior segments were highly active since the number of chaetae in each segment rapidly increases. Thereby, the development of falcigers and spinigers in the posteriormost four segments appear to be more or less balanced in pace. However, the development of falcigers, especially in notopodia, seems to slow down gradually, until in the anterior segments, no notopodial falcigers are formed.

**Fig. 7:**
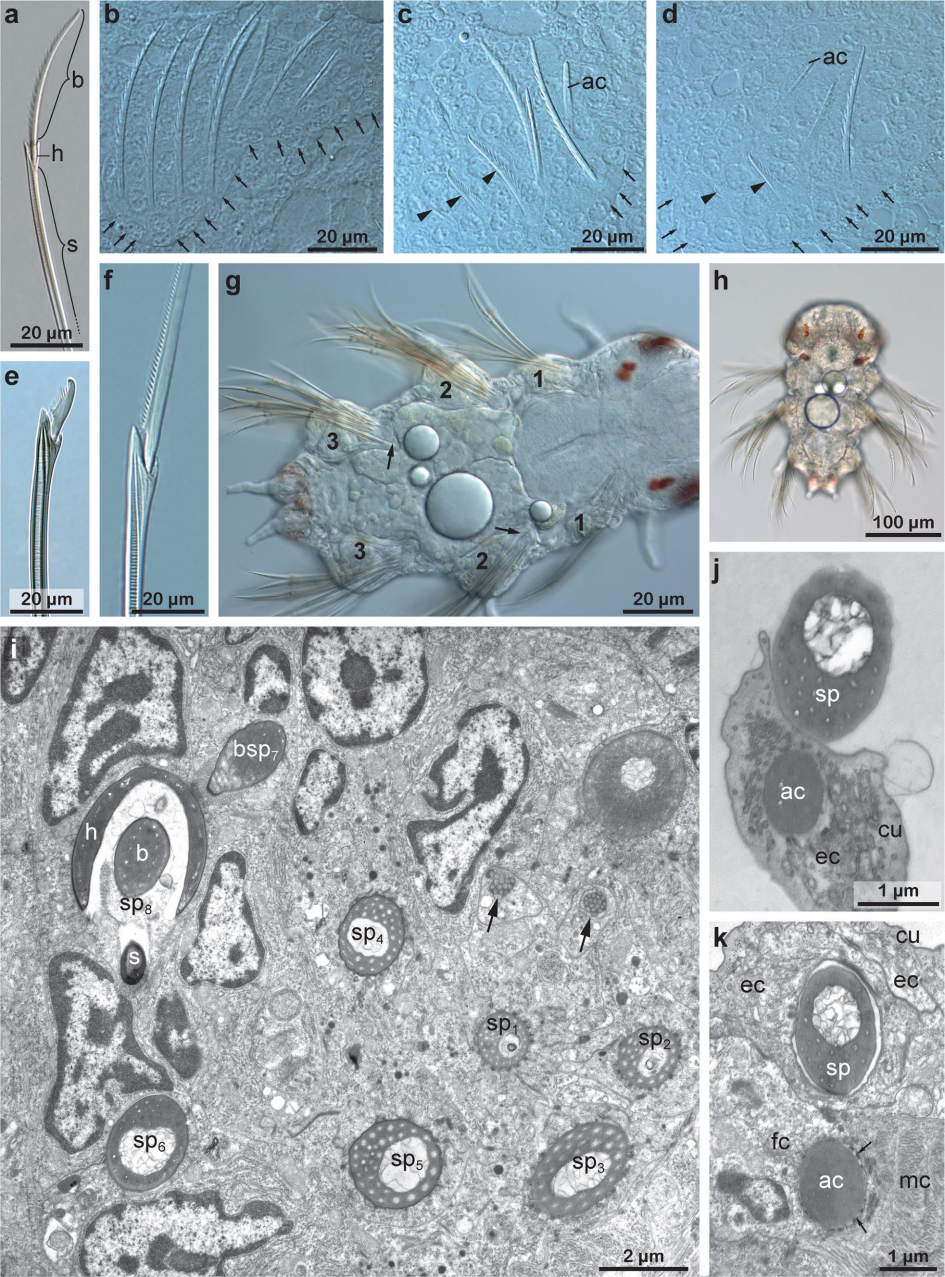
Chaetae and chaetogenesis in 50–104 hpf stages and juveniles of *Platynereis dumerilii*. **a** Spiniger at 80 hpf with serrated blade (*b*), hinge (*h*), and shaft (*s*). **b**–**d** Metatrochophore, 50 hpf. Segmental differences in number of chaetae and ongoing chaetogenesis. Note that none of the chaetae is complete, since a hinge is still not formed. Arrows mark basal ecm of chaetal sac, arrow heads developing blades. **b** Chaetiger 1. **c** Chaetiger 2, **d** Chaetiger 3. **e** Falciger, characterized by a short and stout blade, and **f** spiniger with much longer blade. Note almost identical organization of the hinge. **g**, **h** Nectochaetae at 104 hpf (**g**) and 72 hpf (**h**). Note that all chaetae are spinigers; falcigers are still not formed. Chaetogenesis stops at 72 h in chaetiger 1. The aciculae (*arrows*) are strong in chaetiger 3, weaker in chaetiger 2 and degenerating in chaetiger 1. **i** Chaetiger 2 of a 72 hpf stage. Arrows mark the youngest stages of chaetogenesis. An electron-dense enamel is being formed by releasing elecron dense content from follicle cell vesicles in spiniger 4 and 5. The blade is already formed in spiniger 7 and the hinge has already been formed in spiniger 8. **j**, **k** 72 hpf, the tip of the acicula does not penetrate the cuticle (*cu*), but ends within it. **k** A wider, electron-bright center found in spinigers, is absent in aciculae. Arrows mark hemidesmosomes connecting the intermediate filaments to chaeta. *ec* epidermal cell, *mc* muscle cell, *fc* follicle cell

Overall, the development of chaetae in neuropodia is accelerated compared to that in notopodia. These results also apply to the metatrochophora and the nectochaeta stages which were studied ultrastructurally (Fig. 7).

### Structure of the follicle

**Fig. 8:**
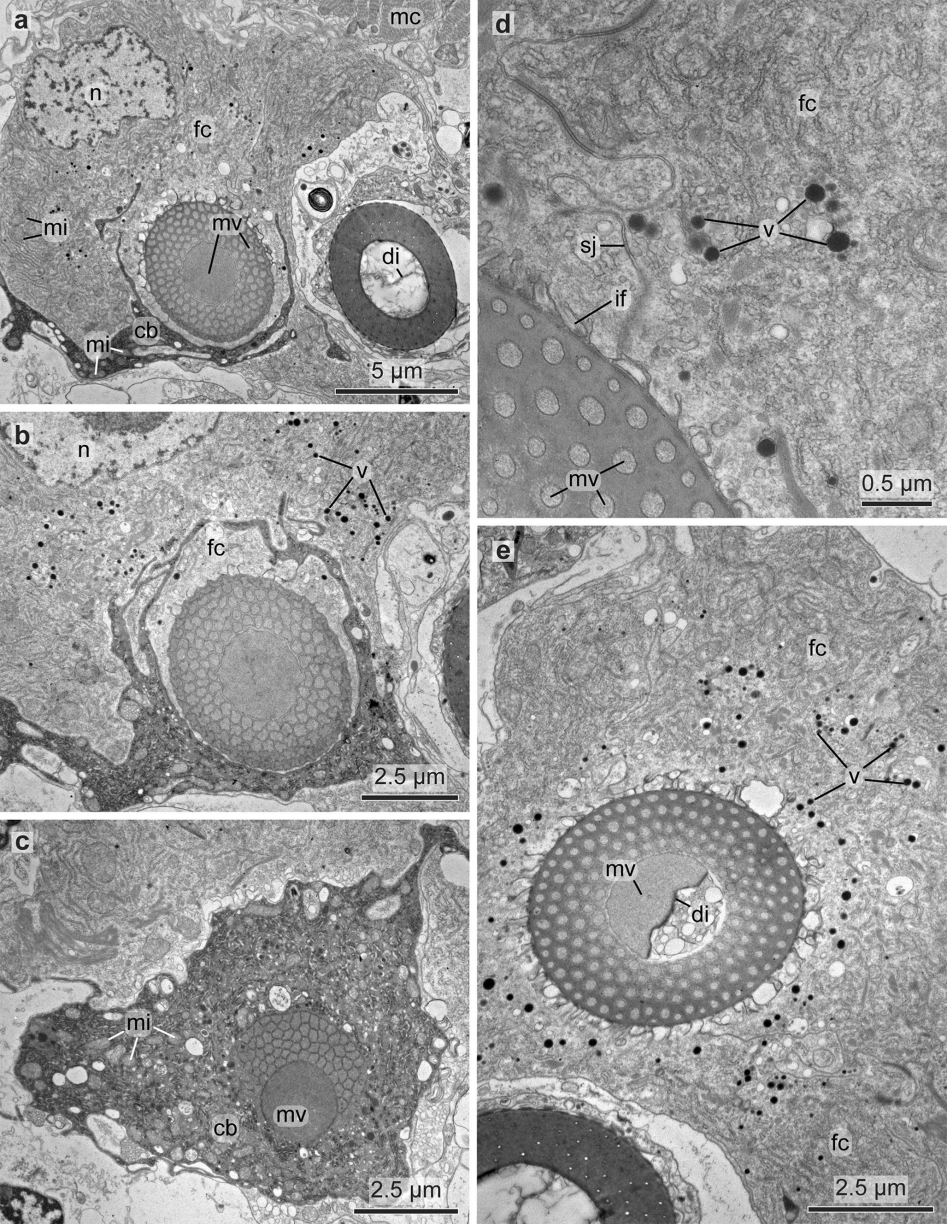
TEM micrographs showing the ultrastructure of the chaetoblast and the chaetal follicle near the chaetal base. **a–c** Chaetogenesis of shaft, note the electron-dense staining of the chaetoblast (*cb*) and the central large microvillus (*mv*) forming the septated canal. *n* nucleus. **d** Close up of two neighboring follicle cells (*fc*) and their septate junctions (sj). Microvilli (*mv*) of the chaetoblast are still visible inside the chaeta, vesicles (*v*) transport the dark chaetal material within the follicle cells. The chaeta is attached to the follicle cells with intermediate filaments (*if*). **e** Note the large number of vesicles. The diaphragms (*di*) in the canal of the chaetal shaft are formed by the large central microvillus of the chaetoblast

**Fig. 9:**
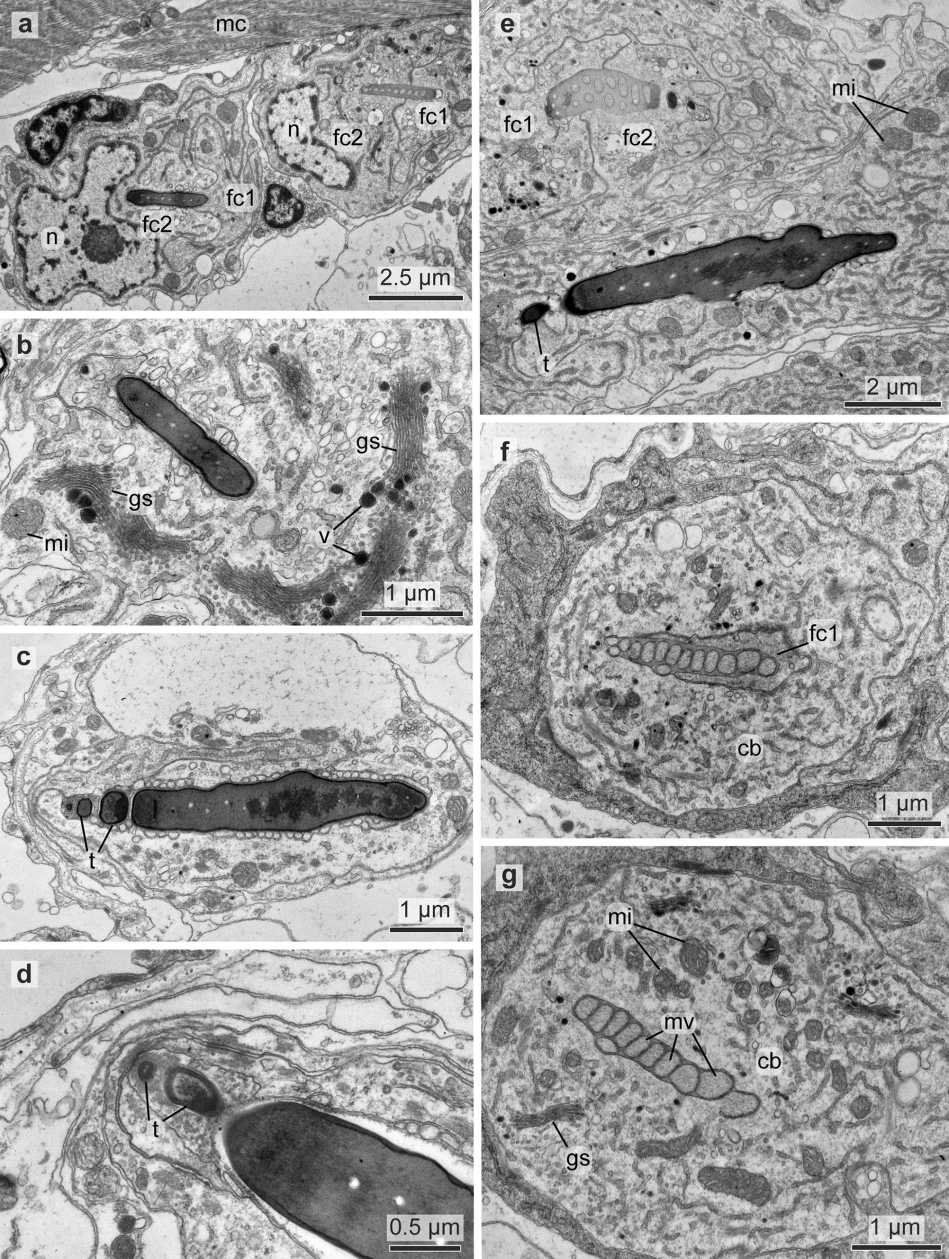
TEM micrographs showing the chaetogenesis of the blade. **a** Two developing spinigers in neighboring follicles. *fc* follicle cells, *cb* chaetoblast. **b** Close up of a follicle cell with electron-dense chaetal material being synthesized by the large number of Golgi stacks (*gs*) and transported in vesicles (*vs*). **c**–**e** Show the development of teeth by addition of a single adrostral microvillus to the row of microvilli (*mv*). **f**, **g** Show the beginning of blade formation with a row of dynamic apical microvilli of the chaetoblast. Note the large number of mitochondria (*mi*) in the chaetoblast. *n* nucleus

Each chaeta and each acicula inserted in its own follicle and each follicle consisted of a basal chaetoblast and several follicle cells. All follicles formed a chaetal sac that is surrounded by an extracellular matrix (ecm) extending the subepidermal basal lamina (Fig. 7b–d). Chaetoblast and follicle cells are epithelial cells that rest on the ecm. Their apical face lined the lumen of the follicle. The chaetoblast was highly branched and intertwined with the follicle cells and neighboring chaetoblasts (Fig. 8a–c). It stained electron-densely, contained numerous vesicles, mitochondria, and a heterochromatic nucleus (Fig. 8c). In compound chaetae, a large central microvillus ranging between 1.2 µm in 72 hpf stages and 2 µm in juveniles and several surrounding rings of smaller microvilli (256 ± 50 nm (*n* = 20)) reached into the lumen of the follicle (Fig. 8c, e). The follicle cells next to the chaetoblast contained numerous vesicles that are released by Golgi-stacks and contained electron-dense stained material (Figs. 8d, e and 9b). These vesicles fused with the apical membrane and released their contents into the small space between follicle and chaeta. In the basal section of the follicle the chaetae acquired an external, electron-dense enamel. Above this section, which is composed of only a few (2–4) follicle cells, these vesicles were missing. The cytoplasm of the follicle cells appeared emptier. Intermediate filaments were crossing the follicle cell and adhered to basal and apical hemidesmosomes that connect the filaments to the ecm and to the chaeta, respectively (Figs. 7i and 8e). The distalmost follicle cells secreted a cuticle that is part of the epidermal cuticle and formed a cuticular invagination when the chaeta was retracted.

The follicle that housed the acicula differs from this description, since it does not pierce the cuticle, but ends underneath the epicuticle (Fig. 7j). Upon mechanical manipulation, however, the tip of the acicula may pierce even this cuticular layer. The acicular follicle originated deep inside the animal and reached through the entire parapodium to ensure its mechanical reinforcement. Neuro- and notopodial aciculae had a prominent dark coloration that faded towards the basis (Fig. 3a, d). In contrast to the other chaetae, the aciculae lacked a septated central canal or any horizontal chambering (Figs. 4b–h and 7i). Numerous microvilli of similar size formed the template of the acicula that persist during the entire lifetime and are not replaced. During growth, additional microvilli were added peripherally to the existing ones and, thus, cause the shape of an elongated cone characteristic for acicula. The acicula extended into the cuticle, but did not pierce it. The follicle cells contained much thicker bundles of intermediate filaments with a strict radial orientation (Fig. 7i). Their ultrastructure underpins their important role as attachment site for the parapodial muscles (Fig. 4a, b).

### Structure of falciger and spiniger chaetae

Both chaetae can be classified as compound chaetae, as a hinge connects their blade-like distal section, to the shaft (Figs. 2c and 7a, e, f). Falciger and spiniger chaetae differed in the size of the blade, in its armor and in the relative position of the hinge (Fig. 7e, f). One side of the blade was armed with a row of short teeth. The armed face of the blade will be called adrostral side; the teeth will be called adrostral teeth. In spiniger chaetae, the blade was slightly helical and up to 602 ± 26 µm (*n* = 7) long (Fig. 7f); in falciger chaetae, it was apically curved, stout, and short, measuring only 27.2 ± 1.4 µm (*n* = 7) in length (Fig. 7e). The blade bore an adrostral row of thick teeth (Fig. 2c), whereas the blade of the former appeared serrated as it bore a neat row of tiny teeth. In epitokous stages, the blade was paddle-like and adrostrally serrated (Fig. 3d).

In falciger chaetae, this hinge was positioned obliquely to the main axis of the chaeta, so that the blade could be deflected into a single direction only. The adrostral edge of the hinge has been called boss by O’Clair and Cloney (1974) (Fig. 2c). Opposite to the boss, the hinge is deeply invaginated allowing for deflecting the blade. A chitinous band, called ligament, prevents unlimited deflection (Fig. 2c). The ligament inserted underneath the boss and underneath the teeth. The shaft contained a large, central, regularly septated canal with horizontal chambering by diaphragms (Figs. 7e, f, and 8a, f).

### Chaetogenesis

In the specimen analyzed we always found two chaetae being formed in neighboring follicles. Formation of one of them was always slightly advanced, so that the course of chaetogenesis could be reconstructed in sufficient detail. Within each follicle, the chaetoblast formed the template for the chaeta. The course of chaetogenesis, however, differed slightly in falcigers and spinigers. Chaetogenesis will be described in four steps.

#### Formation of the blade

In falciger and spiniger chaeta chaetogenesis started within an epidermal invagination that is lined by follicle cells and the chaetoblast. The lumen of the invagination was a narrow compartment that was filled with some electron-grey material. Formation of a row of microvilli on the adluminal surface of the chaetoblast initiated chaetogenesis of spinigers (Fig. 9g), formation of a cluster of microvilli that of falcigers (Fig. 11a). The row consisted of 7 to 10 microvilli in spinigers; the cluster consisted of 15 to 22 microvilli in falcigers. Chitin was released between and peripheral to the microvilli and formed an amorphous electron dense cup, the prospective tip of the chaeta. This material soon stained electron black (Fig. 9e). During the further course of chaetogenesis small microvilli appeared lateral to the existing ones, so that the blade of the spinigers became slightly broader. In falcigers additional microvilli were formed peripheral to the existing group, so that the diameter of the developing chaeta increased. Finally, the tip of the chaeta was completed and defined the rostal end of the chaeta. While basically, a row of microvilli formed the template for the blade of the spinigers the large circular group of microvilli made up the template for the stout tip of the falcigers. Teeth were preformed by single microvilli that merged with the existing cluster or row of microvilli during the course of chaetogenesis (Figs. 9c, d, and 11b). The teeth thus ran oblique to the main axis of the chaeta.

The structure of the spinigers differed slightly in larvae, since they had a slightly thicker, curved and more prominent tip (Fig. 7c). This was also traceable in their chaetogenesis as it started with a bundle of microvilli preforming the tip of the blade instead of a row (Fig. 7i).

#### Formation of adrostal teeth

Adrostally offset, but in line with the existing row of microvilli, repeatedly additional microvilli appeared on the surface of the chaetoblast. Each microvillus was a template for a single adrostral tooth of the blade (Figs. 9c, d, and 11b). It was circular in cross-section (0.35 to 0.4 µm in diameter) in spinigers, but much thicker and broader in falcigers, where it had an oval diameter measuring up to 2.4 µm by 0.85 µm. In both chaetae these additional microvilli were always added adrostally to the existing microvilli. Being offset initially, each additional microvillus was integrated into the row or group of previously formed microvilli during the further course of chaetal formation. Adding teeth initially enlarged the rosto-adrostral diameter of the distal mobile section of the chaeta. Later, the constant merging of microvilli maintained a definitive diameter. In spinigers each new adrostral microvillus was formed after the previously formed one had been integrated into the existing row of microvilli, whereas in falcigers each microvillus was formed prior to integrating the previsously formed one (Fig. 11b). This difference resulted in a serrated adrostal margin of the mobile distal section of spinigers, whereas it caused a staggered row of strong adrostal teeth in falcigers.

#### Formation of the hinge

**Fig. 10:**
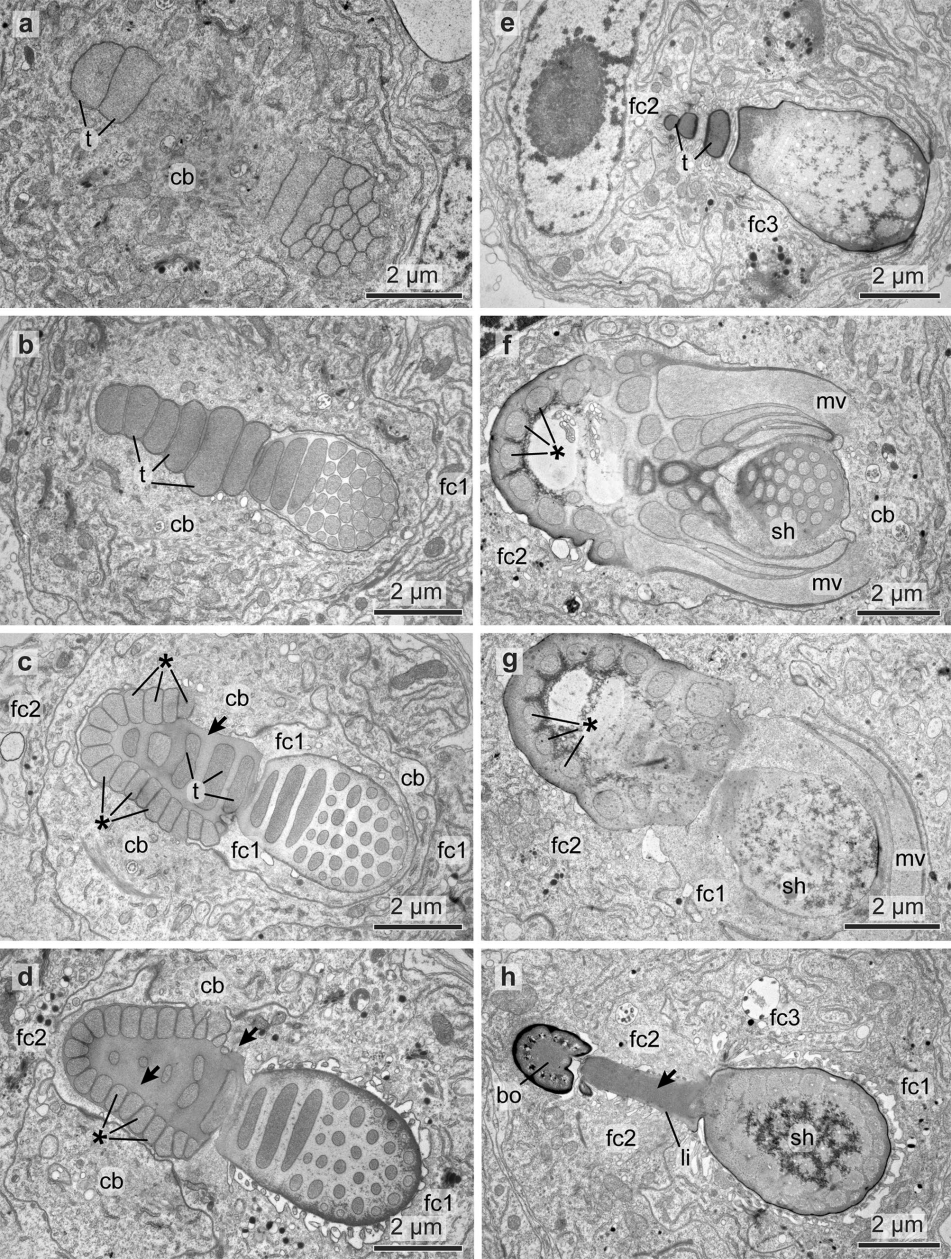
TEM micrographs showing the chaetogenesis of the teeth (*t*), hinge, ligament (*li*), and the apical part of the shaft (*sh*) in a falciger. **a**–**e** Show a series of micrographs through the same developing chaeta, **a** being the basalmost section and **e** the apicalmost. **f**–**h** Show the development of the hinge and ligament in a chaeta, **f** is basal, **h** is apical. * mark the microvilli (*mv*) symmetrically arranged in a horse-shoe shape that form the boss (*bo*). Arrows mark the amorphous chaetal material forming the ligament. *cb* chaetoblast, *fc* follicle cell

The hinge is that region where the movable blade inserts into the shaft (Fig. 2c). It is characteristic for compound chaetae. In spinigers, the hinge consisted of a circular collar formed by the shaft. This collar surrounded the proximal part of the movable apical section. When entering the collar it changed its blade-like shape into a rod-structure that fused in the center of this collar to the shaft (Fig. 10f). In falcigers, the hinge consisted of a much wider collar that was oblique relative to the chaetal shaft. The collar surrounded the blade which was offset from the center of the collar, so that the adrostral section of the collar was wider than the rostral. A chitinous ligament connected the blade to the adrostal part of the collar. The formation of the hinge has been studied in more detail in falcigers.

During formation of the apical section and the adrostral teeth the microvilli sank deeply into the chaetoblast, so that the apical section of the chaetoblast partly surrounded the developing chaeta (Fig. 10c, d). When formation of the hinge started, the apical margin of the chaetoblast was almost on the level of the last teeth formed. An adrostral crescent row of smaller microvilli was then formed by this apical margin (Fig. 10c, d). These microvilli were the template for the boss. Additional microvilli were added rostrally, so that finally, a horse-shoe shaped row of smaller microvilli embrace larger microvilli that were in line with those that once preformed the adrostal teeth (Fig. 10c, d). These were meanwhile integrated into that group of microvilli which preformed the movable apical section. The hinge then consisted of the basal part of the blade and a horse shoe shaped developing boss surrounding a row of larger microvilli.

#### Formation of the ligament

**Fig. 11:**
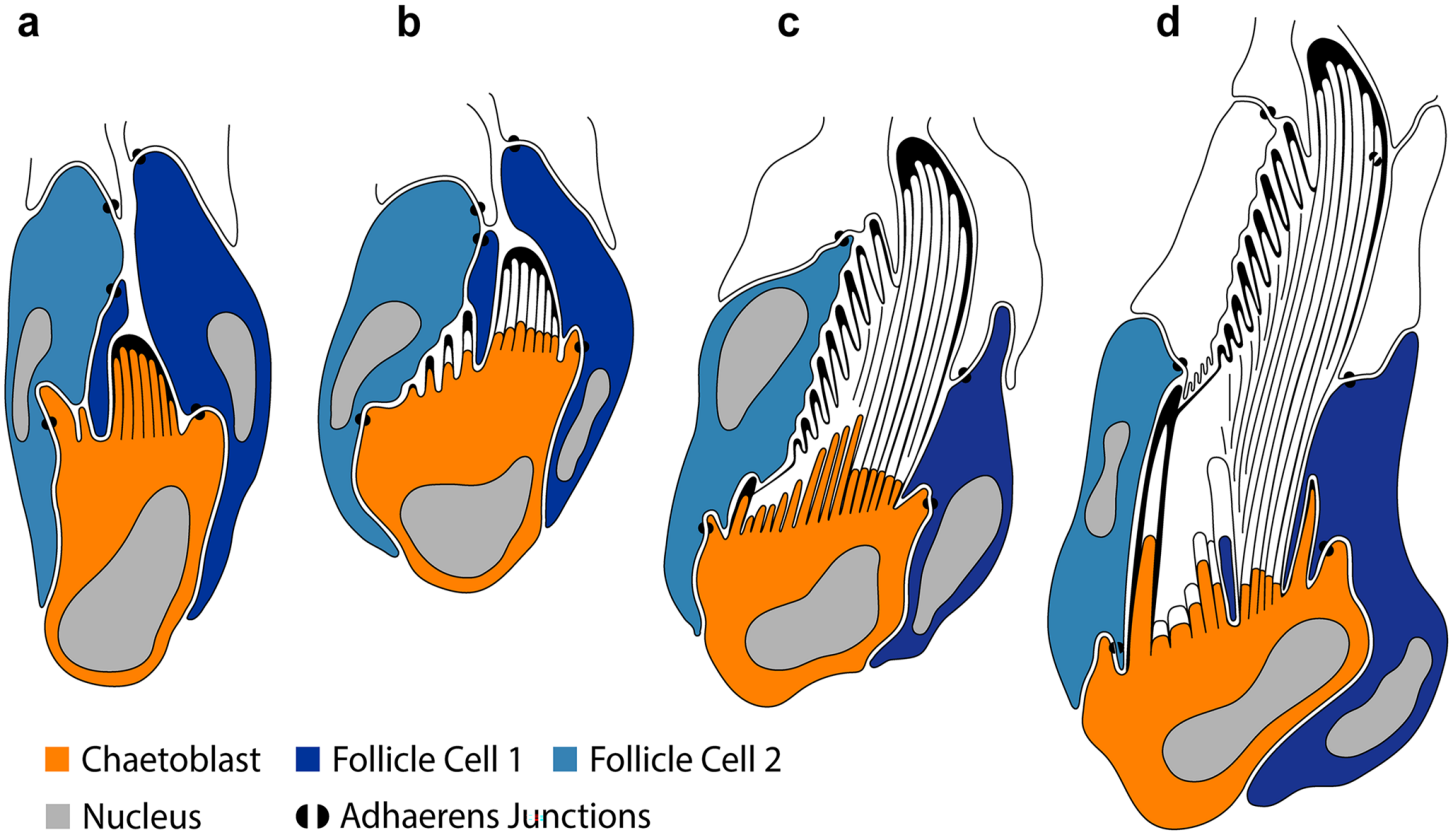
Schematic illustration of chaetogenesis and the interaction between the chaetoblast and the follicle cells as a series of sagittal sections of subsequent representative stages of a developing falciger. **a** Earliest stage of chaetogenesis; formation of the rostrum by a group of dynamic microvilli from the chaetoblast, **b** formation of the teeth by single microvilli, **c** and **d** formation of the ligament and hinge. Note how follicle cell 1 enwraps the group of microvilli that form the base of the blade

A ligament was present in falcigers only. Generally and as described above, the chitinous depositions between the microvilli change their stainability as they become tanned and appear electron darker in electron-micrographs. Often tanning is restricted to the periphery of the microvilli, causing the characteristic hexagonal pattern in transverse sections and refilling of the compartments that remain after the microvillar templates of the chaeta retracted indicate the former position of the microvilli even in fully differentiated chaetae. While forming the ligament, however, this mode of depositing and transforming the chitinous secretions was altered among the larger microvilli that were surrounded by the developing boss and those that once formed the template of the adrostral teeth. There was no tanning and the compartments left by the microvilli were homogenously filled so that their former position could not be inferred from the structure (Fig. 10d, h). These microvilli rapidly retracted, while those of the developing boss and those of the basal part of the blade remained. A follicle cell invaded the space left by the retracting microvilli and a strand of homogenously staining, presumably elastic chitinous band remained, the ligament (Figs. 10g, and 11d). This strand connected the boss to the basal part of the blade; the follicle cell separated the ligament from the surface of the chaetoblast. In the fully developed chaeta, a gap remained where the follicle cell once sat.

#### Completion the hinge region and formation of the shaft

After the ligament had been completed the follicle cell expanded rostrally and surrounded the group of microvilli that preformed the basal section of the blade, thus occupying the space between microvilli and apical section of the chaetoblast (Fig. 10f, g). The follicle cell was filled by a network of actin filaments that seemed to encircle the group of microvilli. By this time the chaetoblast formed a single, huge, crescent microvillus that embraced the abrostal face of the follicle cell that surrounded the basal section of the movable part (Fig. 10g). This microvillus formed the apical rim of the abrostral section of the hinge. The circular arrangement of actin filaments inside the follicle cell diminished the diameter of the group of microvilli preforming the moveable part, causing a small stalk-like flexible element in fully differentiated chaetae. During the further course of chaetogenesis all microvilli merged to form the shaft. The central microvilli fused to form a single large centrally located microvillus. This microvillus then formed the large, central canal with the horizontal chambering of the shaft (Figs. 7e, f, and 8a, f).

## Discussion

*Platynereis dumerilii* is one of the few established model annelids, therefore also an ideal candidate to study chaetogenesis in detail (Zakrzewski 2011; Gazave et al. 2017; Özpolat et al. 2021). This study lays down the necessary descriptive, morphological foundation for further experimental and molecular investigations into this elaborate system.

Interactions among neighboring chaetal follicles, when they are arranged in a row, play a significant role in shaping the final chaeta. This can be observed clearly during the formation of abdominal uncini in *Sabellaria alveolata* (Linnaeus, 1767), where interfollicular cell dynamics directly influence the final shape of a chaeta by tilting the axis of a developing chaeta or by covering parts of the chaetoblast and interfering with the microvillar pattern (Tilic and Bartolomaeus 2016). In other annelids, like Maldanomorpha, where hooks are arranged in a row, ontogenetic changes in chaetal morphology can be observed, where the first hook in a row is not as curved and the bending partially results from interactions between other chaetae developing in neighboring follicles (Tilic et al. 2015). In *P. dumerilii*, interfollicular interactions do not play a significant role in shaping the chaeta. Most important cellular dynamics and interactions occur within a chaetal follicle, for instance when follicle cell 1 enwraps the group of microvilli that form the base of the blade and thereby result in forming the gap that makes up the socket, in which the blade inserts. A similar kind of interaction between follicle cell 2 and the chaetoblast gives rise to the ligament. During these interactions intracellular actin filaments play an important role. Shaping the structure of the chaeta seems to largely depend on modulating the entire intracellular actin filament network which alters the shape and structure of the microvilli and influences cell migration within the follicle.

During formation of the shaft of compound chaetae the actin filaments are much less dominant and are restricted to encircle the developing chaeta. In follicles of the acicula, they are even less prominent; generally, intermediate filaments mechanically connecting these internal chaetae to the body wall muscles dominate the cytoplasm in follicle cells of aciculae. In contrast to the compound chaetae, aciculae are never replaced. They sink inwards during their growth and they do not pierce the cuticle and thus form inner chaetae.

The horizontal chambering of chaetae is characteristic for Nereidiformia, and a synapomorphy shared amongst Chrysopetalidae, Hesionidae, and Nereididae (Pleijel and Gustavsson 2010; Tilic et al. 2019). These internal diaphragms are formed by a large central microvillus that secretes chaetal material discontinuously while repeatedly and rhythmically retracting from the chaeta. This was also observed in the paleae and compound chaetae of Chrysopetalidae (Tilic et al. 2019); the chaetogenesis of chaetae in Hesionidae is yet to be studied. Aciculae, however, never possess an internal chambering and are formed by a uniform microvillar template.

There appears to be an interesting correlation between the length of microvilli and the speed of growth of a chaeta. Longer microvilli are often observed where chaetal elongation occurs rapidly. This is the case in the formation of the long rostal rods of the uncini in *Sabellaria alveolata* (Tilic and Bartolomaeus 2016), where the internal rod rapidly has to grow 80 times the length of the visible chaeta. Similarly, the microvilli observed in the paddle-like chaetae of epitokous *P. dumerilli* also reached a remarkable length of more than 20 µm. This certainly relates to the rapid chaetal turnover and replacement that occurs during transition into the free-swimming epitokous stage.

Our detailed description of chaetogenesis, especially concerning the formation of adrostral teeth, revealed notable differences in the microvillar dynamics than what is described in O’Clair and Cloney’s (1974) pioneering study on chaetogenesis in *Nereis vexillosa*. They interpreted the formation of teeth as the result of a periodic withdrawal of a paraxial group of obliquely flattened dynamic microvilli (Fig. 15 in O’Clair and Cloney (1974)). This is not the case during the chaetal formation in *P. dumerillii,* since each adrostral tooth (both in falcigers and spinigers) is formed by a single microvillus. It seems likely that O’Clair and Cloney (1974) misinterpreted the microvillar arrangement during teeth formation. Such a rapid turning on and off of the microvillar template is highly unlikely and would never result in the observed chaetal morphology. If this were the case, one would expect a row of tiny canals arranged, as microvilli leftovers, in neat little rows inside each tooth. Given that *N. vexillosa* and *P. dumerillii* are both members of Nereididae (Phyllodocida) and are closely related, we expect the same patterns of chaetogenesis during the development of their chaetae in both species.

We also provide a detailed description of hinge (joint) formation. The small microvilli arranged symmetrically in a horse-shoe shape that form the adrostral boss and the single huge crescent-shaped microvillus that builds the abrostal part of the hinge fully demonstrate how complex these microvillar patterns can be that give rise to diverse chaetal morphologies. At this juncture, it is important to note that the formation of the ligament is highly unusual. In contrast to other parts of the chaeta, the ligament is not directly formed by microvillar involvement. The amorphous chaetal material is secreted into a lumen, where it maintains a homogenous and less electron-dense staining when compared with the remaining parts of the chaeta. This is likely necessary for yielding an elastic chitinous structure that allows bending the blade.

Different kinds of compound (or “jointed” chaetae) occur in many polychaete taxa; however, a similar hinge and ligament structure as the one described herein is only known for clades within Phyllodocida (Rouse and Fauchald 1997; Merz and Woodin 2006; Tilic et al. 2016). This type of compound chaeta only has a single ligament, is flexible, and plays a significant role in the locomotory performance of the animal (Merz and Edwards 1998; Hesselberg and Vincent 2006). The sister-group of Phyllodocida, i.e. Eunicida, also has compound chaetae (Tilic et al. 2022). These, however, lack a clear socket and are therefore also referred to as pseudo-compound chaetae with double ligaments (Merz and Woodin 2006; Rouse and Fauchald 1997). The formation of joints and ligaments in Eunicida is yet to be studied. Compound chaetae are also present in Nerillidae, a paedomorphic meiofaunal taxon with unresolved phylogenetic placement (Worsaae 2021) and in some members of Sedentaria like Flabelligeridae and Acrocirridae (Osborn and Rouse 2011). Comparative chaetogenesis has been used to test hypotheses on the homology of certain types of chaetae, like hooks and uncini (Bartolomaeus 1998; Hausen 2005; Tilic et al. 2014; Tilic and Bartolomaeus 2016). This approach is based on the principle that homologous structures are generally expected to be formed by similar morphogenetic processes. This demands a certain amount of structural complexity, which is most definitely the case in joint formation. Therefore, our description of joint formation in a nereidid polychaete will also allow future comparative analyses looking into the formation of joints in other annelids. We expect the joints of Phyllodocida with single ligaments to be an autapomorphy of the group whereas the compound chaetae of Eunicida and Flabelligeridae + Acrocirridae most likely evolved independently. Establishing a well-grounded homology hypothesis for the compound chaetae of Nerillidae might help inform the phylogenetic affinity of the group. A broader comparative dataset on joint formation in annelids is necessary and such analyses will eventually help us to understand the evolution of annelid chaetae, which are critically important in the systematics of the group.

## Data Availability

To allow full transparency of the data presented in this study, all of the aligned serial semi-thin sections used for the 3d-model and the reconstruction of the posterior segments are deposited in https://zenodo.org. Entire image stacks can be downloaded using *Zenodo_get* (Völgyes and Lupton 2020). *Aligned serial sections of the parapodium used for the 3D* - *Model* Direct Link: https://doi.org/10.5281/zenodo.6957608, *Aligned serial sections of the posterior segments* - Direct Link: https://doi.org/10.5281/zenodo.6957628
